# Patterns of cortisol and corticosterone concentrations in humpback whale (*Megaptera novaeangliae*) baleen are associated with different causes of death

**DOI:** 10.1093/conphys/coab096

**Published:** 2021-12-23

**Authors:** Carley L Lowe, Kathleen E Hunt, Jooke Robbins, Rosemary E Seton, Matthew Rogers, Christine M Gabriele, Janet L Neilson, Scott Landry, Suzie S Teerlink, C Loren Buck

**Affiliations:** 1 Department of Biological Sciences, Northern Arizona University, Flagstaff, AZ 86001, USA; 2 Smithsonian-Mason School of Conservation & George Mason University, Front Royal, VA 22630, USA; 3 Center for Coastal Studies, Provincetown, MA 02657, USA; 4 Allied Whale, College of the Atlantic, Bar Harbor, ME 04609, USA; 5 NOAA Fisheries, Alaska Fisheries Science Center Auke Bay Laboratories, Juneau, AK 99801, USA; 6 Glacier Bay National Park & Preserve, Gustavus, AK 99826, USA; 7 NOAA Fisheries, Alaska Regional Office, Protected Resources Division, Juneau AK, 99801 USA

## Abstract

Baleen whales are subject to a myriad of natural and anthropogenic stressors, but understanding how these stressors affect physiology is difficult. Measurement of adrenal glucocorticoid (GC) hormones involved in the vertebrate stress response (cortisol and corticosterone) in baleen could help fill this data gap. Baleen analysis is a powerful tool, allowing for a retrospective re-creation of multiple years of GC hormone concentrations at approximately a monthly resolution. We hypothesized that whales that died from acute causes (e.g. ship strike) would have lower levels of baleen GCs than whales that died from extended illness or injury (e.g. long-term entanglement in fishing gear). To test this hypothesis, we extracted hormones from baleen plates of four humpback whales (*Megaptera novaeangliae*) with well-documented deaths including multiple and chronic entanglements (*n* = 1, female), ship strike (*n* = 2, male and female) and chronic illness with nutritional stress (*n* = 1, male). Over ~3 years of baleen growth and during multiple entanglements, the entangled whale had average corticosterone levels of 80–187% higher than the other three whales but cortisol levels were similar to two of the other three whales. The nutritionally stressed and chronically ill whale showed a slow increase in both cortisol and corticosterone spanning ~3 years, followed by a sharp decline in both hormones before death, possibly indicative of adrenal failure in this moribund individual. This whale’s correlation between cortisol and corticosterone was significant but there were no correlations in the other three whales. Our results show that cortisol and corticosterone concentrations vary according to the type and duration of illness or injury. Single-point GC concentrations should be interpreted with caution as low values can occur in whales experiencing pronounced stress and individual baselines can be highly variable. Baleen analysis is a promising tissue type for retrospective analyses of physiological responses to various stressors affecting baleen whales.

## Introduction

Large whales are increasingly being exposed to a variety of stressors, including increases in ocean noise (e.g. vessel traffic, military sonar, seismic gas and oil exploration), ship strikes, entanglement in fishing gear, marine heatwaves (Suryan *et al.,* 2021) and prey shifts (Thomas *et al.*, 2016). However, determining how stress affects physiology has been proven difficult due to the inherent challenges with assessing the physiological state of free-swimming cetaceans. Extended monitoring and repeated sampling is exceedingly challenging in these large-bodied, highly mobile species that spend the majority of their time underwater (Hunt *et al.,* 2013). Physiological state is most commonly assessed via blood sampling, but that requires animals to be handled (in captivity or by trapping), neither of which are feasible for baleen whales. Non-traditional sample media have instead been used to inform physiological state, including earplugs (Trumble *et al.*, 2013; Trumble *et al.,* 2018) or baleen (Hunt *et al.*, 2017a; Hunt *et al.*, 2017b; Fernández Ajó *et al.,* 2018, Hunt *et al.,* 2018; Fernández *et al.,* 2020) from dead whales. In live whales, measures of stress response have been obtained from feces (Hunt *et al.,* 2006, 2019; Pérez *et al.,* 2011; Hunt *et al.*, 2014a; Corkeron *et al.*, 2017; Rolland *et al.,* 2017; Valenzuela-Molina *et al.*, 2018), respiratory vapour (Burgess *et al.*, 2018; Mingramm *et al.*, 2019) or blubber (Pallin *et al.*, 2018; Teerlink *et al.*, 2018; Mingramm *et al.*, 2020).

Physiological responses can be caused by stress from natural (e.g. predation, reproduction) and anthropogenic (e.g. entanglement, ship strike) causes. The relative influence of these stress responses can be gauged via measurement of glucocorticoids (GCs) (Romero, 2002). GCs are involved in the modulation of behavioural and physiological responses and are mediators in the recovery of a stress response (Sapolsky *et al.*, 2000). After encountering a stressor, GCs are released from the adrenal cortex, helping animals mobilize energy stores by breaking down glycogen in the liver, diverting blood flow to exercising muscles, stimulating immune function and reducing nonessential activities such as digestion, growth, tissue maintenance and reproduction to help re-establish homeostasis after encountering a stressor (Reeder and Kramer, 2005; Lattin and Romero, 2014; Sapolsky *et al.*, 2000). Two GCs involved in this stress response and recovery, cortisol and corticosterone, are synthesized and secreted by all mammals but which of these two is the ‘dominant’ GC (i.e. more abundant hormone in plasma) differs among species. Almost all mammals are cortisol dominant while most non-mammals, as well as rodents, are corticosterone dominant (Koren *et al.,* 2012). Mysticetes have traditionally been assumed to be cortisol dominant, but recent data (Hunt *et al.*, 2017a; Fernández Ajó *et al.,* 2018; Lysiak *et al.*, 2018) indicate that this may not be the case and that the two hormones may respond differently depending on the stressor.

Since stressors can differ in duration, GCs can be elevated for varying lengths of time, making it important to use tissue types that reflect the time frame of the study question. Respiratory vapour (‘blow’), feces, blubber, baleen and earplugs all capture differing durations of adrenal activity and have various latency patterns. Blow provides a non-invasive measure of short-term physiological changes and most likely reflects circulating blood levels (Hunt *et al.,* 2014a; Hogg *et al.,* 2009; Burgess *et al.*, 2018; Mingramm *et al.*, 2019), while fecal hormone concentrations generally reflect average endocrine activity over the preceding 1–2 days (4–48 hours; Rolland *et al.*, 2005; Hunt *et al.,* 2013). Blubber steroid hormone concentrations generally reflect short-term endocrine activity, from hours (Champagne *et al.,* 2018) to weeks (Kershaw and Hall, 2016), and can be useful for sampling live whales (Pallin *et al.*, 2018; Dalle Luche *et al.*, 2019; Mingramm *et al.*, 2020). Earplugs contain waxy, keratinaceous layers that are deposited throughout the whale’s entire lifetime, but they are difficult to recover intact from most carcasses and the resolution for hormone analysis is limited to ~6 months (Trumble *et al.*, 2013; Trumble and Usenko, 2015; Crain *et al.,* 2020; Trumble *et al.,* 2018).

Baleen is an especially promising sample type for long-term stress and reproductive analysis because hormones are incorporated into the keratinous baleen plate as it grows (Hunt *et al.,* 2016, 2017b, 2018; Fernández *et al.,* 2018, 2020). Since baleen grows slowly and continuously over many years, a single baleen plate can hold a retrospective longitudinal record at approximately a monthly or bimonthly resolution, depending on sampling distance, of the concentrations of GCs in the whale over the baleen growth record. For mysticetes with shorter baleen (e.g. humpback whales), this period is 3–5 years versus a decade or more in species with longer baleen (e.g. bowheads, *Balaena mysticetus*) (Aubin *et al.*, 1984; Lubetkin *et al.*, 2008; Lysiak, 2008; Bentaleb *et al.,* 2011; Ryan *et al.,* 2013). In this study, we sought available US specimens of humpback whale baleen with a known cause of death. Four such specimens were found: two cases of ship strike, one case of chronic illness and one case of entanglement. The GC concentrations along the baleen plate were quantified to determine if hormone patterns correspond with end-of-life events. Specifically, we asked the following two questions: (i) ‘Do baleen cortisol and corticosterone concentrations differ among whales that died following extended entanglement, ship strike or illness?’ and (ii) ‘Do cortisol and corticosterone concentrations co-vary through time?’ If so, sampling one hormone might be sufficient to determine physiological responses, but if not, it would be important to understand how these two hormones vary in concentration with stressor type and if it is necessary to quantify both. We addressed these research questions by quantifying cortisol and corticosterone concentrations along the length of a single baleen plate from four humpback whales, each of which represents ~3 years of growth.

## Materials and methods

Museum databases, National Oceanographic and Atmospheric (NOAA) stranding databases and other scientific entities were queried for baleen of humpback whales with known causes of death. The baleen had to be available for destructive sampling which is not possible for some older, historical baleen plates. The Arctos database was queried and provided results from 197 collections; 9 of these plates were from humpback whales but none had known causes of death. A Smithsonian Natural History Museum database query resulted in 21 humpback whale baleen plates, but none that were available for sampling had known causes of death. Glacier Bay National Park & Preserve (AK, USA) and the National Oceanographic and Atmospheric Administration Protected Resources Alaska Division (AK, USA) were contacted about baleen plates from humpback whales with known causes of death and those queries resulted in three North Pacific humpback whale plates included in this study. An additional plate from the North Atlantic was provided by the Center for Coastal Studies (MA, USA) and College of the Atlantic (Bar Harbor, ME, USA). Baleen, in all of these cases, was recovered during necropsy by members of the US Marine Mammal Standing Network.

### Study animals

The four available plates represented four adult humpback whales with well-known life histories and documentation of the events surrounding their deaths, as described below and summarized in Table 1.

**Table 1:** Background information for four humpback whales, including sex and age, cause of death (COD), and average concentrations of corticosterone and cortisol with number of samples (n) in nanograms of hormone per gram of baleen powder

Whale ID	Stranding ID (Institution)	Sex (age)	COD	Location	Avg Corticosterone ± SD (ng/g) (n samples)	Avg Cortisol ± SD (ng/g) (n samples)
SEAK 68	AK-2001038 (NOAA Alaska Region)	F(44.5)	Ship strike	Point Gustavus, Alaska	1.78 ± 0.36 (24)	1.84 ± 0.60 (25)
SEAK 1536	AK-2018046 (NOAA Alaska Region)	M(≥35)	Ship strike	Admiralty Island, Alaska	2.55 ± 1.70 (32)	3.27 ± 1.20 (32)
Spinnaker	COA150611Mn (Center for Coastal Studies)	F(11)	Entanglement^a^	Cape Cod, Massachusetts	5.11 ± 1.50 (33)	1.51 ± 0.57 (33)
SEAK 441	AK-2016094 (NOAA Alaska Region)	M(66)	Illness	Icy Strait, Alaska	2.84 ± 1.51 (29)	1.62 ± 1.05 (29)

Spinnaker’s age was known based on sighting records as a calf, while the age of SEAK 1536 was a minimum age based on previous sighting records. SEAK 68 and SEAK 441’s ages were determined via earplug analysis. The number of corticosterone and cortisol samples vary due to the amount of powder recovered from the baleen at each location.

^a^Proximal cause of death.

SEAK 68 (F) was first reported in the Alaska fluke catalogue in 1975 in Glacier Bay, Alaska, and was subsequently sighted in Alaska and Hawai’i 11 times (Gabriele *et al.*, 2010); she had a calf in five of her sighting years and was pregnant at death (fetus length, 39.2 cm; Gulland, 2001). She was found dead in 2001 in Glacier Bay, Alaska, after being struck by a cruise ship and based on her condition and blubber thickness, she appeared to be in good health at the time of death. An earplug analysis indicated that she was 44.5 years old (Gabriele *et al.*, 2010).

SEAK 1536 (M) was estimated to be a minimum of 35 years old based on his first sighting in 1983 as a non-calf (Adam A. Pack, University of Hawaii at Hilo Marine Mammal Laboratory). He was found dead near Point Young in Southeast Alaska in 2018 and was sighted once in Southeast Alaska in July 2017 (Heidi Pearson, unpublished data). Extensive haemorrhaging, including significant haemorrhage and gelatinous bruising around the cranial dorsolateral abdomen and copious blood in the abdominal cavity, along with skull fractures, suggest ship strike as the most likely cause of death (Savage, 2018). Blubber depth was noted as normal, and body condition (subjective determination of nutritional status/health made by a veterinarian) was rated between average and good (Savage, 2018).

Spinnaker (North Atlantic Humpback Whale Catalog #8587, F) was first catalogued as a calf in the Gulf of Maine, the southernmost primary feeding ground in the North Atlantic. By the time of her death in 2015, she had been involved in four documented entanglements in fishing gear and at least three additional events inferred from injuries that are diagnostic of entanglement. In 2006, at the age of 2 years, Spinnaker was found entangled in fishing gear and was successfully disentangled by the Atlantic Large Whale Disentanglement Network. Despite the severity of that entanglement and associated injuries, she was responsive upon release and thought likely to recover. Spinnaker was subsequently re-sighted with new entanglement injuries in 2009, 2012 and 2013, but the exact timing and mechanism of those entanglements are unknown. In September 2014, Spinnaker was witnessed entangled again and another formal disentanglement operation was mounted. Disentanglement responders documented a complicated entanglement involving several sets of fishing gear entangling and injuring multiple body parts. In the end, the only piece of gear that could not be removed was a short section of gillnet emerging from her mouth. As in 2006, her behaviour at release was considered positive and highly responsive. Spinnaker had been seen the day before, and so it was known that the majority of this life-threatening entanglement had occurred within the previous 24 hours. However, a review of images from the prior day confirmed that the gillnet portion in the mouth had already been present for an unknown period of time. There were no subsequent sightings of Spinnaker until May 2015, at which time she was seen with the same gillnet as well as new entangling rope running through her mouth and around the base of her tail leading to heavy fishing traps, effectively anchoring her in place. A disentanglement response resulted in the successful removal of all gear apart from the portion in her mouth. However, her condition at release was assessed as poor and her behaviour was unresponsive, except for an adverse reaction when efforts were made to remove the gear from the mouth.

Spinnaker was found dead in June 2015, at which time it was possible to more thoroughly study her entanglement and injuries. During necropsy, it was found that, in addition to other injuries, entangling gear had cut 27 cm deep into the bones of her rostrum, completely severing the vomer (the small bone separating the left and right nasal cavities) and splitting both the left and right upper jawbones (Seton *et al.*, 2015). The margins of these skeletal lacerations had indications of bone regrowth, indicating that these injuries did not occur at a time close to death (Seton *et al.*, 2015), although the exact timing of the injuries was not known. Spinnaker was 11 years old when she died, with no reproduction-related stressors known to have contributed to her history (i.e. was never pregnant; Lowe *et al.,* 2021). However, her length at the time of death was only 1079 cm (Seton *et al.*, 2015), which is small for a female of her age in this population (e.g. Stevick, 1999) and all well-documented sightings during the study period indicated below-average body condition with injuries still in the process of healing.

SEAK 441 (M) was first sighted near Juneau, Alaska, in 1972 and died in 2016 in Southeast Alaska, making him one of the longest sighted humpback whales on record (Gabriele *et al.,* 2021). His necropsy showed a relatively high concentration of whale lice on the skin, scattered superficial erosions and ulcerations suggestive of generalized debilitation or immunosuppression, multiple tissue infections and multiple severe caudal vertebrae abnormalities (Gabriele *et al.,* 2021). These ailments are thought to be the result of a prolonged illness that was likely exacerbated by nutritional deficiencies caused by broad ecosystem level impacts from the 2014–2016 extreme North Pacific heatwave (Arimitsu *et al.,* 2021; Suryan *et al.,* 2021); measurement of blubber depth during necropsy indicated nutritional stress with poor body condition and low lipid content in the blubber. SEAK 441 was aged using earplug analysis and was found to be 66 years old (Gabriele *et al.,* 2021).

### Measurement of baleen plates and estimating date of growth

Baleen was removed from the carcass by cutting the plate at gumline. If necessary, baleen was cleaned by soaking in freshwater and then the plate was laid flat to dry. Baleen plates were measured every centimetre with a tape measure applied and marked along the posterior face of each plate ~2 cm from the labial edge (Hunt *et al.*, 2016). The base of the plate (near gumline, newest baleen) was designated as the ‘0 cm’ point and was assigned an estimated growth date of the day before the whale was found dead.

Baleen for the whales was analysed for estimation of growth rate of the baleen plate via counting the number of annual cycles in ratio of ^15^N/^14^N (δ15N) (Lowe *et al.,* 2021; Rogers *et al.,* 2021). This ratio changes seasonally, as whales migrate between isotopically distinct summer and winter prey (Hobson & Schell, 1998; Hobson *et al.*, 2004; Lubetkin *et al.*, 2008). However, there is variation in migration timing both within and between individuals; therefore, stable isotopes can only yield an estimate of annual growth in baleen. Each year was determined from δ15N peaks after smoothing from four nearest neighbours.

### Pulverization of baleen and extraction of hormones

Hormone extracts were prepared using a hand-held electric rotary grinder (Dremel Model 395 Type 5) to abrade a short (~1.5 cm) transverse groove across the posterior face of the plate, starting at the ‘zero point’, with baleen powder collected on a piece of weigh paper. Successive samples were drilled every 2 cm along the complete length of the plates (total: 27–33 samples per plate). Hormones were extracted from samples with 4.00 ml of 100% methanol added to 75.0 mg of homogenized baleen powder, followed by 2 hours of vortexing, centrifugation, recovery of 3.00 ml of supernatant and dry down (Hunt *et al.*, 2016). Extracts were reconstituted in 0.50-ml assay buffer (#X065 buffer used for both assays on the manufacturer’s recommendation; Arbor Assays, Ann Arbor, MI, USA), sonicated for 5 minutes, shaken for 5 minutes, transferred to cryovials, cooled and then decanted to new cryovials to remove any remaining particulates. All extracts were stored at −80°C and assayed for GCs within 1 year. Due to the natural tapering of the baleen plate, not all samples had enough powder to be included in the analysis. Missing values are from either a powder sample that was <50.0 mg due to the width of the plate or degradation of the plate at that location (*n* = 8). SEAK 68 was sampled for other studies (Lowe *et al.,* 2021) so cortisol was sampled every odd centimetre and corticosterone was sampled every even centimetre.

### Hormone assays

For both hormones, samples were diluted to 1:4 dilution using X065 assay buffer. The order of samples in the plate was randomized both within and across assays to reduce influence of inter- and intra-assay variation. Samples with a coefficient of variation (CV) of >10%, outside 5–95% binding or 100% or more from their nearest neighbour were re-diluted as necessary and re-assayed. Nonspecific binding wells and blanks (zero dose) were assayed in quadruplicate with all standards, controls and samples assayed in duplicate. Results were converted to picograms of immunoreactive hormone per gramme of powdered baleen.

Cortisol was quantified with a commercially available enzyme immunoassay (EIA) kit, which has previously been validated for baleen extracts of humpback whales, as well as eight other species of baleen whales (catalogue #K003-H1, Arbor Assays, Ann Arbor, MI, USA) (Hunt *et al.,* 2017b). The manufacturer’s protocol has six standards spanning 100–3200 pg ml^−1^, an additional low-dose standard was created by mixing equal volumes of 100 pg ml^−1^ sample with X065 assay buffer for a total of seven standards. Assay methods were conducted according to the manufacturer except standards were created in the same buffer used to prepare the powdered extracts (X065 assay buffer), based on the manufacturer’s recommendation (Hunt *et al.,* 2014b). The sensitivity limit is 45.4 pg/ml, average intra-assay precision is 8.8% and average inter-assay precision is 8.1%.

Corticosterone was also quantified using an EIA (catalogue #K014-H1, Arbor Assays, Ann Arbor, MI, USA), which has been validated for humpback baleen in previous studies (Hunt *et al.,* 2017a, 2018). The corticosterone EIA has six standards spanning 78.125–10 000 pg ml^−1^ according to the manufacturer’s protocol; an additional low standard was added by diluting the lowest standard by 1:2 to capture low values that were seen in pilot studies. The sensitivity limit for pooled baleen corticosterone in baleen whales (H*unt et al.*, 2017b) is 16.9 pg/ml, average intra-assay precision is 5.2% and average inter-assay precision is 7.9%.

## Statistical analysis

Correlations between cortisol and corticosterone were compared in each whale with Spearman’s correlation test; SEAK 68 was not analysed for correlation because sampling for each hormone was offset by 1 cm. Significance threshold was set at an alpha of 0.05. Data analysis was performed with GraphPad Prism 8.1.1.

## Results

### Baleen growth rates based on stable isotopes

**Figure 1:**
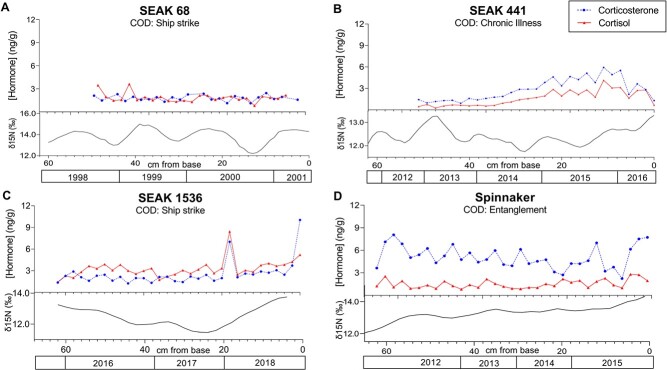
Corticosterone (blue circles) and cortisol (red triangles) for four humpback whales (*Megaptera novaeangliae*) with various causes of death: ship strike (**A** and **C**), chronic illness (**B**) and entanglement (**D**). The x-axis shows the measurement in centimetres from base of the baleen plate (i.e. newest baleen = 0 cm) with y-axes showing concentration of hormone in ng/g of dried powder and δ15N. Lower graphs show δ15N peaks after smoothing from four nearest neighbours; note the different ranges between panels. Year is determined retrospectively from date of death and stable isotope analysis and is an estimate only.

**Figure 2:**
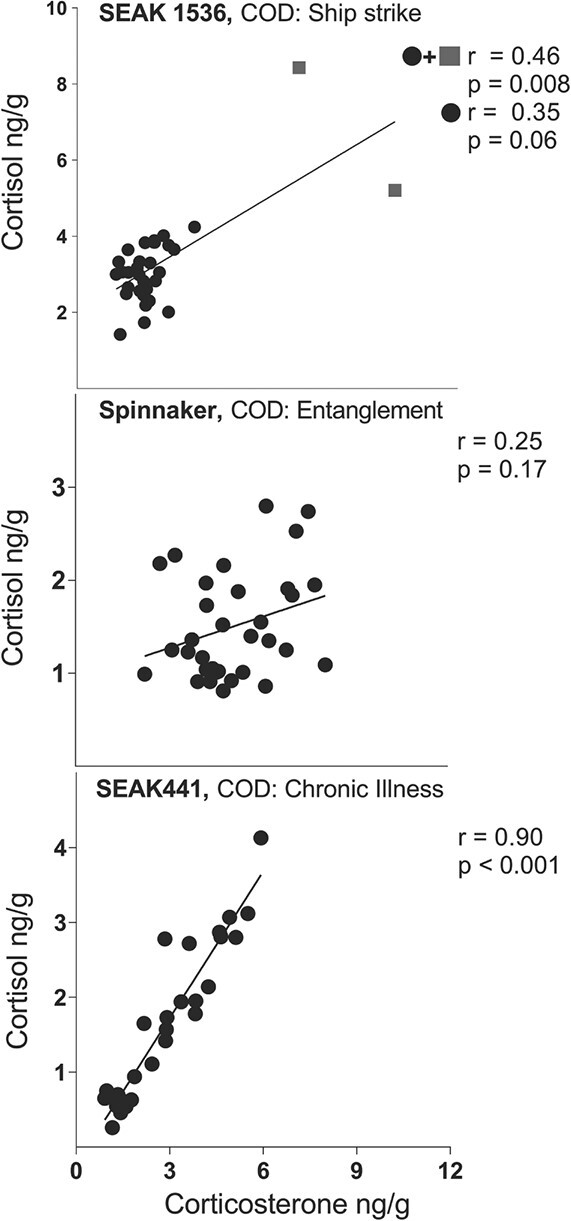
Relationship between cortisol and corticosterone for three humpback whales (*M. novaeangliae*). Pearson’s correlations are provided for each individual whale. SEAK 1536 (**A**) was killed by ship strike; SEAK 441 (**B**) died from a long-term illness and nutritional stress; and Spinnaker (**C**) died after repeated and long-term entanglements in fishing gear. Units are nanograms of hormone per gramme of baleen powder. Top panel (SEAK 1536) shows correlation with two outliers (grey squares) as well as without the outliers (black circles). COD, cause of death.

For SEAK 68 (F, 44.5 years old), three full annual cycles were apparent from SI data with partial additional cycles, yielding a duration of growth that spanned an estimated 3.5 years (Fig. 1). SEAK 68’s average annual baleen growth rate (BGR) was 17 cm (three full years of growth at 20, 16 and 15 cm; Fig. 1). For SEAK 441 (M, 66 years old), baleen growth based on SI data was slightly slower, with an average annual BGR of 12.75 cm (four full years of growth at 9, 12, 15 and 15 cm, Fig. 2). SEAK 1536 had an average BGR of 18.3 cm/year (three full years of growth at 20, 18, 17 cm/year) while Spinnaker had an average BGR of 19 cm/year (only two full cycles were apparent, at 20 and 18 cm). These BGRs are within the range documented for other North Pacific humpbacks (Matt Rogers, unpublished data).

### Temporal patterns of GCs—ship strike cases

The two ship-struck whales had different patterns of GC concentrations from each other, with the 44.5-year-old female (SEAK 68) having relatively stable GC baleen concentrations compared to the ≥35-year-old male (SEAK 1536) (Fig. 1, panels A and C). SEAK 68 had two spikes in cortisol during the older baleen growth (~97% and 88% increase above average) but there were no large spikes in corticosterone (Fig. 1, panel A). SEAK 1536 had large cortisol and corticosterone spikes (157% and 175% above average, respectively) at 18 cm (~1 year before death; Fig. 1, panel C). He also showed high levels of both hormones at 0 cm, the newest baleen growth just prior to his death, with a 293% increase above average of corticosterone and a 61% increase in cortisol. His average overall cortisol concentration was 78–117% higher than the other three whales (Table 1).

### Temporal patterns of GCs—entanglement case

Using an average of 20 cm/year for baleen growth, it was determined that Spinnaker’s growth period covered ~3 years, a period when she experienced repeated and prolonged entanglements in fishing gear. She had an average corticosterone that is 80–187% higher than the other three whales (Table 1; Fig. 1, panel D). In the two samples taken from the baleen growth period during known entanglements, her corticosterone levels peaked to 31% and 51% higher than the full plate average; however, cortisol levels did not also spike during this time. In 2014, Spinnaker’s corticosteroids rose abruptly and rapidly declined, possibly in response to the majority of the entangling gear being removed. This pattern is also consistent with her positive behaviour after release, despite the severity of her entanglement and the fact that she already carried a portion of entanglement that ultimately led to her death. At ~4 months before death (winter 2015) corticosterone increased rapidly from relatively low levels (~56% lower than average) and quickly climbed until death, when her corticosterone was 51% higher than her total plate average at the 1 cm sampling location. There are no independent data to determine what specifically triggered the onset of that final peak but her failure to return to baseline is consistent with her poor prognosis at the time of release and the nature of injuries detected after death. Less well understood is the peak in the oldest baleen growth (61 cm), at which time corticosterone peaked at 58% higher than average, her highest level for the entire plate. A sighting in June of that year revealed that Spinnaker had unhealed injuries from a recent, unreported entanglement, but the injuries were similar in nature and severity to those routinely documented among free-swimming whales in this population (e.g. Robbins and Mattila, 2001; Robbins, 2012) and others (Neilson *et al.*, 2009; Basran *et al.,* 2019; Ramp *et al.,* 2021). Although her corticosterone levels were still much higher than the other whales, her average cortisol (1.505 ng/g) was comparable to SEAK 68 (1.844 ng/g, ship strike death) and SEAK 441 (1.618 ng/g, chronic illness; Fig. 1).

### Temporal patterns of GCs—nutritional stress/chronic illness case

SEAK 441, the nutritionally stressed 66-year-old male, had a gradual, simultaneous increase in both GCs over ~3 years of baleen growth, until a sharp decline in both GCs ~6 months before death (Fig. 1, panel B); the older half of the plate averaged 200% less cortisol and corticosterone than the newer half of the plate (containing end of life growth).

### Relationship of cortisol to corticosterone

The cortisol:corticosterone correlation was unique for each whale (Fig. 2); SEAK 68 was not examined for correlation as the sampling for cortisol and corticosterone were offset from one another by 1 cm and could therefore not be directly compared. The three whales analysed showed varying strengths in the correlations between the two hormones, with a statistically significant correlation seen in only one individual, the ill and nutritionally stressed male, SEAK 441 (*r* = 0.90, *P* < 0.001).

Cortisol dominance was found in all but two sampling points in SEAK 1536. SEAK 68 had varying cortisol and corticosterone dominance throughout the plate while SEAK 441 and Spinnaker had corticosterone dominance with all corticosterone concentrations being higher than cortisol concentrations throughout the entirety of the baleen plate.

## Discussion

We assessed humpback whale physiological responses to three stressors over multiple years preceding death by analysing baleen GC concentrations in tandem with life history events from sighting records. The re-creation of life histories was made possible by long-term humpback whale photo-identification studies in Southeast Alaska and the Gulf of Maine. The addition of life history events through photo identification allowed for factors such as body condition, calving events and behaviors to be linked to physiological information. Though data are at present limited to four case studies, these cases demonstrate notable differences in cortisol and corticosterone concentration patterns among whales that suffered from ship strike, illness or long-term entanglement prior to death. The unique stress responses that we observed were as follows: a concurrent stepwise increase in both cortisol and corticosterone followed by a sharp decline prior to death for a nutritionally stressed and chronically ill mature male, low and stable GCs for a pregnant whale that died from a ship strike, two large spikes in corticosterone and one spike in cortisol in a male that died from a ship strike, and consistently high corticosterone but low cortisol in a nulliparous female who experienced multiple entanglements in fishing gear.

### Ship-struck whales

We examined two humpback whales that died from ship strikes and found large differences in GC concentrations between these individuals, both of whom were from the North Pacific population. The 44.5-year-old female (SEAK 68) had relatively stable and low GCs throughout the baleen plate. She was never seen entangled and had one calf during the baleen growth period (and was pregnant when she was killed), demonstrating that she was healthy enough to maintain pregnancies. The other individual was a ≥35-year-old male (SEAK 1536) whose cortisol concentrations averaged twice those of the other whales. His cortisol and corticosterone concentrations both spiked for one sampling point ~1 year and then 1 month prior to death. His histology report noted that he had bone remodelling that could be indicative of a previous injury (Kathy Burek, personal communication) but the timing of this injury is unknown. Both spikes correspond with a period of broad ecosystem level impacts that persisted following the 2014–2016 North Pacific marine heatwave (Suryan *et al.,* 2021). His feces also tested positive for low levels of domoic acid and abraxis (Kathy Burek, personal communication) and it is possible that the spike in GCs 1 year prior to death was from exposure to these or similar algal toxins associated with the marine heatwave causing neurological dysfunction or other physiological disturbances that may even have predisposed him to being struck.

The pregnant female showed no increase in GCs just prior to death, strengthening the likelihood of an immediate death in accordance with the necropsy report that concluded ‘the extent of the skull damage would have been immediately fatal’ (Gulland, 2001). However, the spike in GCs seen in the male just prior to death could suggest either that he did not immediately die after being struck by the ship or that he experienced an injury or other condition that increased the chances of being struck by a ship. Though ship strike cases are often assumed to represent acute mortality only, with no chronic stress before death, some whales do survive the ship strike for some period afterwards (weeks or longer) (Moore *et al.*, 2004). Furthermore, it is possible that whales are more likely to be struck by a ship if they are already impaired in some way, i.e. prior injury or disease that impedes the whale’s ability to detect or avoid ships. However, in this study it is assumed that the ship-struck whales had relatively quick deaths based on necropsy information showing good physical health of each whale (average body condition and normal blubber thickness; Gulland, 2001; Savage, 2018). The effect of a fatal ship strike on GC concentrations in recently grown baleen will likely depend on previous physiological condition and the time it takes for the whale to die. To our knowledge, no other studies have examined baleen GC concentrations in whales killed by ship strike. However, fecal GCs in North Atlantic right whales (*Eubalaena glacialis*) showed higher GCs in individuals that were entangled or stranded versus struck by a ship (Rolland *et al.,* 2017).

### Entangled whale

The entangled whale (Spinnaker) had corticosterone levels that were two times higher than the other whales. Elevated corticosterone has been observed in the baleen hormone profiles of entangled individuals from other species including North Atlantic right whales (Hunt *et al.,* 2018; Lysiak *et al.*, 2018) and bowhead whales (Rolland *et al.*, 2019). Fecal GCs in North Atlantic right whales have also been shown to be significantly higher when entangled or stranded compared to ship-struck or healthy whales (Rolland *et al.,* 2017). Elevated GCs have additionally been found in blubber samples from cetaceans that stranded, were entrapped or entangled (Kellar *et al.,* 2015; Trana *et al.*, 2016; Champagne *et al.,* 2018; Mingramm *et al.*, 2020). GCs can increase at times of heightened energetic needs (Sapolsky *et al.*, 2000; Sapolsky, 2002). An increase in GCs could occur during entanglement if swimming, breathing and feeding is impaired. Additionally, entanglement involves injury, tissue damage and pain, all of which elicit a stress response (Moore and Van der Hoop, 2012). Our study confirms that entanglements can also induce elevated GCs in humpback whales (as evidenced in their baleen) and is the first study to examine GC patterns resulting from multiple entanglement events. The finding that corticosteroids were elevated throughout Spinnaker’s baleen record is consistent with her repeated history of life-threatening entanglement and her apparent health prior to and during the study period. Indeed, continuous exposure to entanglement may help explain her below-average body length, as has recently been documented in North Atlantic right whales (Stewart *et al.,* 2021). It is noteworthy that two of three major peaks coincide with reported, life-threatening entanglement events. However, this study also suggests a major event in early 2012 that was missed by observational data. The outward injuries that were observed in June 2012 were consistent with those observed frequently among free-ranging humpback whales in the Gulf of Maine. This suggests that some of these apparently minor injuries may be more biologically significant than previously assumed, just as the full severity of injuries to Spinnaker’s jaw could not be ascertained from her live sightings. Individuals in populations in which entanglements and other stressors are common may exhibit higher baseline stress and respond differently than individuals in more pristine populations (Madliger and Love, 2014). Larger sample sizes from individuals with known histories will improve understanding of the physiological responses of humpbacks within and among populations.

### Nutritionally stressed and chronically ill whale

The nutritionally stressed whale’s (SEAK 441) cortisol and corticosterone concentrations gradually increased in tandem until 6–8 months prior to death and then fell to low levels until death. The necropsy revealed extensive bone malformations, excessive whale lice and overall poor body condition (Gabriele *et al.,* 2021). Because these issues occurred over an extended period, it is likely that his rising GCs reflect when the stressor began and suggest that he suffered from this issue for up to 2 years. The sharp decline in both GCs just prior to death could indicate that he experienced adrenal fatigue during the final stages of starvation. During this period of baleen growth (2012–2016), the North Pacific marine ecosystem experienced a severe heatwave from 2014 to 2016 that resulted in increased sea surface temperatures (anomalies greater than +3°C; Cavole *et al.,* 2016, Di Lorenzo and Mantua, 2016) and weakened coastal upwellings that could have contributed to this whale’s nutritional stress. A similar rise-then-fall pattern in GCs has been documented in baleen of southern right whale (*Eubalaena australis*) calves with chronic wounding from kelp gulls (Fernández Ajó *et al.,* 2018; Fernández Ajó *et al.,* 2020). In these calves, baleen GCs gradually increased as wounding became more severe, and then abruptly decreased to baseline or lower immediately before death. Longitudinal patterns in GCs are therefore important to determine, as a single-point sample near the end of life could suggest the nutritionally deficient whale had low GC content and would lead to the spurious conclusion that the animal was not stressed, when in actuality he was likely experiencing adrenal collapse prior to death, as seen in the gull wounded calves.

### Corticosterone vs cortisol covariation

The four whales in our study exhibited different temporal covariation between cortisol and corticosterone. Of the three whales available for statistical analysis between the two hormones, one of the whales showed a significant relationship, one showed a visual trend between the two hormones and the other whale showed no relationship. Corticosterone has been less studied in marine mammals because it has not historically been considered the ‘dominant’ GC (i.e. the most abundant hormone in plasma). However, little data exist to support the assumption that baleen whales are cortisol dominant. No baseline plasma GC concentrations have ever been obtained from unstressed baleen whales; rather, the few plasma hormone studies available used samples obtained from individuals that were hunted or live-stranded (Kjeld, 2001; Rolland *et al.,* 2017). Such acute-stress events often alter GC ratios; for example, cortisol in terrestrial mammals typically responds faster to acute stress than does corticosterone (Koren *et al.,* 2012). We showed cortisol dominance throughout the baleen of the younger male humpback whale and at various points throughout the baleen growth in the older female humpback while the two other whales had corticosterone dominance. This pattern is inconsistent with a previous study that showed a male bowhead, a male North Atlantic right whale and a male blue whale (*Balaenoptera musculus*) had corticosterone dominance throughout most of their baleen growth (Hunt *et al.,* 2017a). In a North Atlantic right whale calf baleen, cortisol was higher than corticosterone, but the baleen of southern right whale calves showed variability in the dominant hormone (Hunt *et al.,* 2017a). Together, these findings suggest the potential for varying GC dominance in baleen whales but emphasize the need for additional studies examining both GCs in various tissue types.

It is possible that cortisol and corticosterone may not only react differentially to stress, but also stimulate slightly different physiological responses (i.e. ‘dual glucocorticoid signaling’; Koren *et al.,* 2012). For example, the entangled female had corticosterone levels twice as high as the other whales, but her average cortisol levels were the lowest of the four whales in our study. This could imply that entanglement of humpback whales might cause high levels of corticosterone, but not cortisol, although more individuals with similar life histories should be sampled to further explore this pattern. It is also possible that corticosterone and cortisol vary with stress duration and severity. In a study of mice, both cortisol and corticosterone increased on Day 1 of stress (using repeated or unpredictable acute and chronic stressors), but afterwards cortisol levels remained stable while corticosterone dramatically declined during repeated restraints (Gong *et al.,* 2015). In another rodent (*Ctenomys talarum*), cortisol responded to fasting but corticosterone did not (Vera *et al.*, 2019). These studies highlight that cortisol and corticosterone are not strictly interchangeable hormones due to their different secretion responsiveness to stimuli. Further sampling and analysis are needed to determine whether cortisol and corticosterone have a consistent relationship in healthy whales and whether the two hormones respond differently to acute versus chronic stress.

Using known individuals from well-studied populations with documented life history events is paramount when beginning studies on interpretation of physiological health. Baleen plates from known individuals hold important retrospective information about how physiological conditions relate to confirmed behaviours, migration patterns, reproductive state and body conditions prior to death. Current methods of baleen analysis allow for each individual to be intensively sampled, making it possible to evaluate multiple aspects of animal physiology and stress over past years or even decades, depending on the species. However, it is rare for baleen to be recovered from whales with sighting histories, making it more important to collect tissue samples from animals with known sighting histories. We encourage researchers to take advantage of the extensive photo-identified populations available (e.g. North Atlantic right whales, North Atlantic and Pacific humpback whales) before extrapolation of physiological limits to the population level in groups that do not have sighting records to assist in confirming reproductive or life history state. As more tissue types and individuals are sampled and become validated in multiple baleen species, it is likely that these techniques can then be applied to baleen whale populations that lack sighting history records.

## Conclusions

To conserve and promote population recovery of baleen whales it is necessary to understand how these whales react to natural and anthropogenic stressors. Baleen and earplugs can provide important retrospective analysis of stress hormones while blubber, blood or blow can provide insight into real-time changes in physiological state. As modern whale populations encounter increased noise, entanglement, pollution and marine heatwaves in their environments, it is important to have the ability to quantify stress responses to mitigate stress levels, especially in endangered and threatened populations. It is vital to incorporate time series analyses as the duration and magnitude of the GC release can more accurately portray an individual’s physiological condition. Earplugs should also be collected when possible since they can be used to determine age and to evaluate lifelong hormone profiles, although with less fine temporal resolution than that of baleen (Trumble *et al.*, 2013; Trumble *et al.,* 2018; Crain *et al.,* 2020). Single samples of cortisol and corticosterone to determine stress responses, regardless of tissue type, should be interpreted with caution; low values can occur in whales experiencing pronounced stress, particularly moribund individuals that are experiencing adrenal exhaustion, and individual baselines can be highly variable.

Ship strikes and entanglements are currently recognized as some of the primary threats to baleen whale populations (Thomas *et al.*, 2016), suggesting that to implement effective policy change it is necessary to understand these impacts, as well as their relative and combined contributions. This could be elucidated using retrospective techniques such as the methods used in this study. Our results demonstrate the profound physiological response that results from continuous entanglement. These entanglements, and entanglement risk, surely have detrimental consequences even when death is not the final result and may contribute to chronic stress. Not only can GC analysis inform on acute versus chronic causes of death, but it could also help point to earlier events in the lives of the whales that may have predisposed them to entanglement or ship strike. Routine baleen collection and analysis could clarify causes of death and help inform management strategies with the goal of reducing anthropogenic-caused mortality events. The methods used here, in conjunction with nonlethal sampling techniques (e.g. blubber biopsy, drone body condition, fecal and blow analysis), could assist in delineating short-term acute stress events versus long-term chronic stressors that could be sublethal or lethal and help inform both individual and population level health.

## Funding

The laboratory work was supported by research funding from the Technology and Innovation Fund at Northern Arizona University, Flagstaff, AZ, USA.

## Permits

Baleen was collected under NOAA permits 18 786, 932-1489 and 932-1905-MA-009526 and/or authorized for study under NOAA Marine Mammal Parts Authorizations issued to the Center for Coastal Studies, Stranding Agreement issued to Allied Whale of the College of the Atlantic and K.E. Hunt.
